# WITHDRAWN: The effect of ischemia on expression quantitative trait loci (eQTL) in human myocardium and insights into myocardial injury etiology

**DOI:** 10.21203/rs.3.rs-3967889/v1

**Published:** 2024-02-20

**Authors:** Azam Yazdani, Sameeksha Tiwari, Mahyar Heydarpour

**Affiliations:** Harvard Medical Center; Harvard Medical Center; Harvard Medical Center

**Keywords:** ischemia, expression quantitative trait loci (eQTL), myocardium, myocardial injury etiology

## Abstract

To understand the pathological processes of myocardial ischemia in humans, we performed RNA sequencing of the left ventricle (LV) tissue samples in 118 patients undergoing aortic valve replacement surgery at baseline and after cold cardioplegic arrest/ischemia, single-cell RNA sequencing was additionally performed in four patients. We characterized the genetic basis of interindividual variation in the transcriptome of human LV in baseline and post-ischemia conditions by the identification of local expression quantitative trait loci (cis-eQTL). We also conducted a genome-wide association study in an independent cohort of 2,371 patients undergoing coronary artery bypass graft surgery to assess the association between genetic variants and myocardial injury. We integrated the results with the eQTL data and identified the causal genes of myocardial injury. Finally, using mouse ischemic data, we assessed the similarity with human LV transcription in genes differentially expressed at the two conditions.

The cis-eQTL were replicated with high rates in both internal and external cohorts. We did not observe any dramatic change in the impact of cis-eQTL on gene expressions in baseline condition compared to post-ischemia condition. We identified 10 eQTLs with putative causal effect on troponin as a biomarker of myocardial injury (*p*-value < 0.005), such as *TYW1, USP49, FLG, TMEM80, and GBAP1,* which were differentially expressed in human data (*p*-value < 8E-3) whereas *TYW1* and *TMEM80* were also differentially expressed in mouse data (*p*-value < 0.01). We observed the higher expression of most causal genes in cardiac myocytes at post-ischemia condition, however, *CFAP161* with a causal effect on troponin (*p*-value = 0.002) had a higher expression in endothelial cells. *CFAP161* and two other causal genes *MRAS* and *ICA1L* (p-value < 0.02) shared regulatory loci with myocardial infarction using external data. The findings in this study provide insights into eQTL changes due to ischemia-induced during bypass surgery, a major clinical problem. Since this type of ischemia shares commonalities with MI, the findings may provide insights into the pathological processes of myocardial ischemia and offer potential clinical applications.

## Introduction

Ischemic heart disease is a major source of perioperative morbidity and mortality. Myocardial ischemia occurs when there is a mismatch between coronary oxygen delivery and metabolic requirements of the myocardium. In the human heart, the damage of myocardial ischemia begins after approximately 20 to 40 min of oxygen deprivation and may lead to cell death if the ischemic result is severe^1^. Genetic variants associated with myocardial infarction (MI) risk have been found in genome-wide association studies (GWAS)^2–5^. However, little is known about the biological and functional mechanisms by which these variants cause ischemic heart disease.

Ischemia may regulate the effect of a genetic variant on a molecular pathway, and, ultimately, on the disease condition^6,7^. The most proximal trait to a genetic variant is gene expression. Due to the cis effect of single nucleotide polymorphism (SNP) alleles on transcription and translation gene expression quantitative trait loci (eQTL), some eQTL are disease-context dependent, observable only under specific physiological conditions, e.g., unique stress or stimulus. eQTL shows a high degree of tissue specificity^8–10^, emphasizing the need to examine disease organ tissue. Therefore, we studied patients experiencing myocardial ischemia while undergoing cardiac surgery with cardiopulmonary bypass (CPB). CPB, although induced by cold cardioplegic arrest and not coronary occlusion, is associated with obligatory ischemic myocardial injury evidenced by increased cardiac biomarker release, and therefore, shares commonality with ambulatory MI.

We performed whole-transcriptome next-generation RNA sequencing of the left ventricle (LV) tissue samples in 118 patients undergoing elective aortic valve replacement surgery with CPB before and after the obligate ischemia during aortic cross-clamping (TRANSCRIBE cohort). We performed cis-eQTL analysis in this cohort, i.e., genetic variants located proximal and associated with gene expression of a given gene. In addition to the TRANSCRIBE cohort, we had a separate set of 2,371 patients undergoing coronary artery bypass graft surgery (CABG genomics cohort), which in troponin was recorded, a complex of three regulatory proteins that is integral to muscle contraction in cardiac muscle and elevated levels in blood after cardiac surgery. We performed a GWAS with myocardial injury and integrated the data from the TRANSCRIBE cohort into the CABG genomics cohort to provide insights into myocardial injury etiology. We also performed an enrichment analysis using external data from CARDIoGRAMplusC4D Consortium11. On a subset of individuals from the TRANSCRIBE cohort, we performed single-cell analysis and provided the cell types with the maximum number of reads mapped on the genes identified in different analyses performed here. Finally, using a mouse model, we assessed the similarity in human transcription with mouse data in gene expression.

## Results

We profiled the transcriptomes of LV tissue of 118 patients from the TRANSCRIBE cohort before the onset of ischemia (baseline sample) and again shortly before the removal of the aortic cross-clamp (post-ischemia sample). Associations between variants from genotyping and gene expression levels were examined to map the eQTL, association of genome-wide genotyping and transcriptomics. The 7eQTL mapping was performed for baseline and post-ischemia conditions. We focused on cis- (local) eQTL. False Discovery Rate (FDR) was applied to account for the large number of tests between variants and expressed genes in each condition; eQTL with FDR below 0.05 was considered significant. In each condition (baseline and post-ischemia), we identified significant eQTL which include eGene and eSNP.

**Figure 1:**
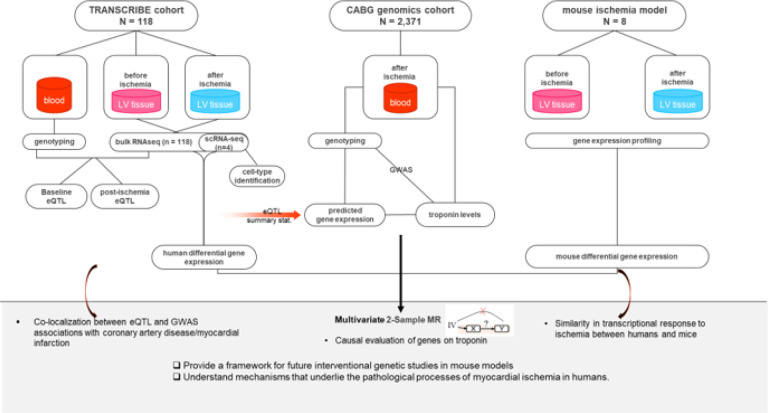
Study design and analysis workflow. In the TRANSCRIBE cohort, 118 tissues were sampled twice, before and after exposure to ischemia of cardiac surgery. *Cis*-eQTL were mapped within baseline and post-ischemia conditions. Out of 118 samples, 4 randomly selected samples underwent single-cell RNA sequencing (scRNA-seq) and were used for cell type identification. We also performed differential gene expression analysis on both bulk RNA-seq and scRNA-seq data. In an independent cohort of 2,371 patients undergoing cardiac surgery (the CABG genomics cohort), we performed GWAS on troponin levels, as a biomarker of myocardial injury. The eQTL summary statistics from the TRANSCRIBE cohort were used to predict gene expression levels in the CABG genomics cohort. While predicting expression levels, the MR assumptions were satisfied. Therefore, the causal effects of gene expressions on myocardial injury were investigated. Using the eQTL summary statistics of the TRANSCRIBE cohort and GWAS summary statistics of the CARDIoGRAMplusC4D Consortium on coronary artery disease (CAD) and myocardial infarction (MI), we performed colocalization analysis to determine whether identified eQTL and CAD/MI GWAS share common genetic loci, indicating a potential causal relationship. Differential gene expression analysis was performed in human and mouse ischemic data and the similarity was assessed.

We also carried out a GWAS in an independent cohort of 2,371 cardiac surgical patients (CABG genomics cohort) to assess the association between genetic variants and myocardial injury (defined as an increase in cardiac troponin levels). Using the eQTL summary data from the TRANSCRIBE cohort, we predicted the expression levels in the CABG genomics cohort. While predicting the gene expression levels, the Mendelian randomization (MR) assumptions were satisfied. The prediction provided us with an opportunity to assess the causal effect of gene expressions on myocardial injury. In addition, we identified variants sharing regulatory (eQTL) and disease-associated signals with coronary artery disease (CAD) and MI performed on the external data from CARDIoGRAMplusC4D Consortium^11^. For 4 randomly selected samples out of 118 in the TRANSCRIBE cohort, we performed single-cell RNA sequencing in both conditions and provided the results of differential expression analysis as well as cell types with the maximum number of counts (number of reads mapped on a given gene) for the findings in this study. Finally, we explored the similarity of transcriptional response to ischemic injury in humans and mice by comparing differentially expressed genes. The study design and analysis workflow are reviewed in Fig. 1, and the details of the materials and analyses are explained in the Methods section.

### Genetics of gene expression at baseline and post-ischemia conditions in human LV myocardium.

**Figure 2:**
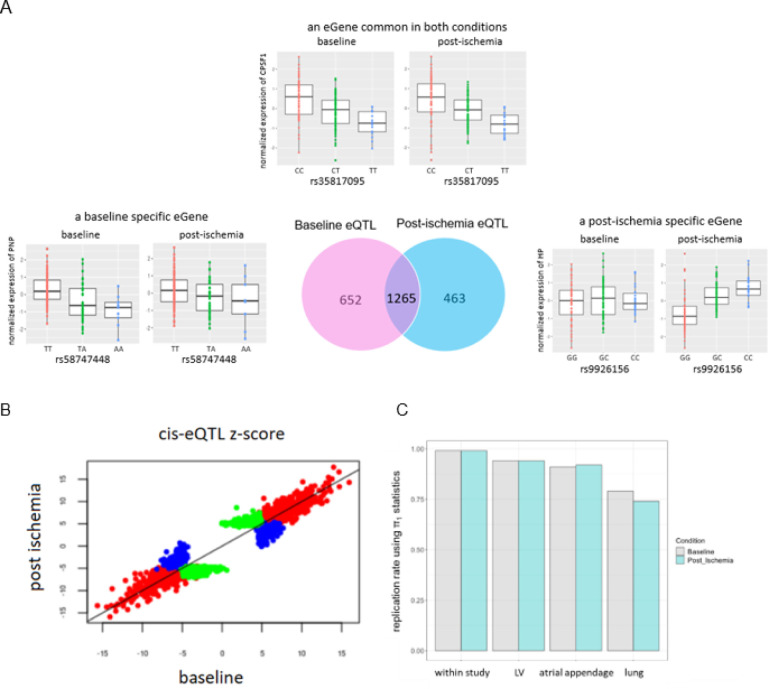
Ischemia cis-eQTL study in human LV myocardium. **A**. **A** Venn diagram representing the number of genes with at least one significant cis-eQTL (FDR < 0.05) at baseline and post-ischemia conditions. From each of the three categories common eGene, baseline-specific eGene, and post-ischemia-specific eGene, one eGene is randomly selected and the boxplots of the normalized expressions in baseline and post-ischemia conditions are depicted by genotypes of the top cis-eQTL (smallest *p*-value), (x-axes). The center line corresponds to the median, and the lower and upper hinges indicate the 25^th^ and 75^th^ percentiles. The highest and lowest dots show the maximum and minimum gene expressions. **B**. Comparing the effect sizes of top cis-eQTL in baseline and post-ischemia conditions. The large effect sizes belong to eGenes common in both conditions (red dots). The smaller cis-eQTL effect sizes belong to either baseline-specific eGenes (blue dots) or post-ischemia-specific eGenes (green dots). The effect sizes were distributed around the 45-degree line, which means the effect sizes do not change dramatically after exposure to ischemia, as expected. There is no dot in either the upper left or lower right quadrants of the plot, which means no cis-eQTL changed the direction of effect between the two conditions. **C**. Replication of baseline cis-eQTL in post-ischemia and vice versa (within the study), as well as replication of baseline and post-ischemia cis-eQTL in GTEx Heart tissues (LV and atrial appendage) as well as lung tissue (a non-heart tissue). The lower rate of replication in non-heart tissue shows that the high rate of replication in heart-related tissues is not by chance.

To characterize the genetic basis of interindividual variation in the transcriptome of human LV myocardium in both baseline and post-ischemia conditions, we mapped expression Quantitative Trait Loci (eQTL) in the TRANSCRIBE cohort, (Fig. 1). We focused on cis-eQTL, where gene expression levels are associated with variants located in the 1-Mb flanking the gene of interest. We identified cis-eQTL for 1,917 (12.8% of tested genes) and 1,728 genes (11.5% of tested genes) with FDR ≤ 0.05 in baseline and post-ischemia conditions respectively, (Supplementary, Tables S1–2). Hereafter, we use ‘eGenes’ to refer to genes with at least one significant cis-eQTL. In total, 1,265 genes (8.4% of tested genes, 53% of eGenes) had an eQTL in both conditions; of these, 446 eGenes (more than 35% of shared eGenes) had the same most significant variant. By comparing baseline eGenes with post-ischemia eGenes, we found condition-specific eGenes, genes being eGenes only in one of the two conditions. Of 1,917 baseline eGenes, 652 were baseline-specific (34.0% of baseline eGenes and 27.4% of total eGenes), and of 1,728 post-ischemia eGenes, 463 were post-ischemia-specific (26.8% of post-ischemia eGenes and 19.5% of total eGenes), (Fig. 2A and Supplementary, Tables S3–5). The condition-specific eGenes had smaller cis-eQTL effect sizes compared to the eGenes common in both conditions, (Fig. 2B). The effect sizes did not change dramatically after exposure to ischemia and no eQTL effects changed allelic direction between the two conditions, (Fig.2B).

We assessed the replication of cis-eQTL using several datasets, (Fig. 2C). First, the replication of baseline cis-eQTL in the post-ischemia condition and vice versa was conducted. Replication was quantified using the π1 statistic^12^, with very high replication rates, π1 = 0.99 for replication of both conditions. Second, we replicated our eQTL with eQTL from the Genotype-Tissue Expression (GTEx) project^13^. The π1 estimates of our top eQTL *p*-values for both baseline and post-ischemia conditions were 91–94% in GTEx Heart tissues (left ventricle and atrial appendage) and 74–79% in GTEx lung tissue (a non-heart tissue), which is lower than the replication rate in the heart tissues and represents the high rate of replication in heart tissues is not by chance.

### Identification of eGenes with a role in the etiology of myocardial injury.

To test whether interindividual genetically-based variation in the transcriptional response to ischemia might contribute to interindividual variation in myocardial injury, we performed a two-sample multivariable MR technique with genes as explanatory variables/exposures and troponin as an outcome.

**Figure 3:**
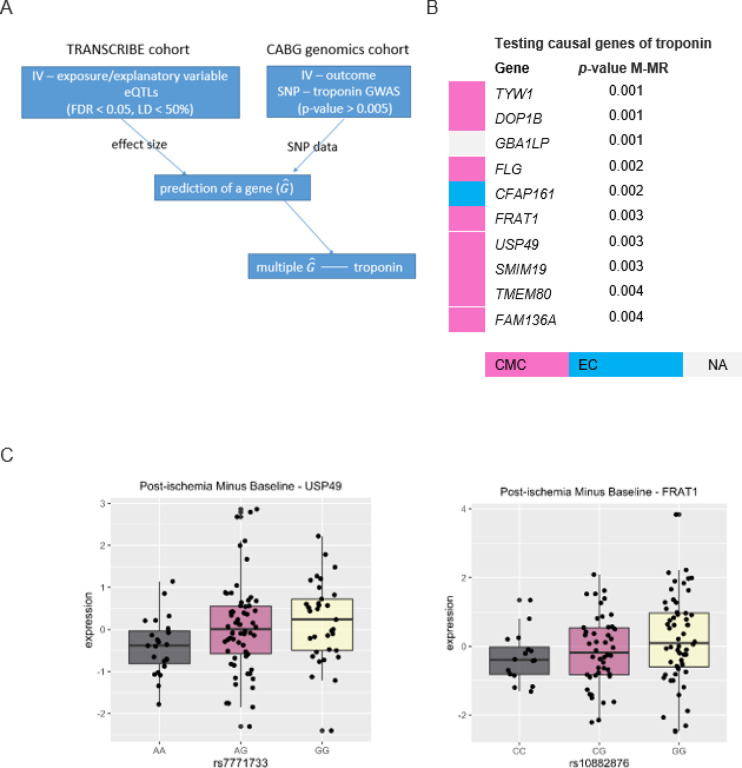
Causal effect of eGenes on troponin, a biomarker of myocardial infarction (MI). **A**. An algorithm for a two-sample MR technique^14^ with eGenes as explanatory variables/exposures and troponin as an outcome. The eQTL summary data from the TRANSCRIBE cohort and genotyping individual-level data from the CABG genomics cohort were used to predict the post-ischemia eGene levels in the CABG genomics cohort. For each eGene, multiple eQTL (FDR < 0.05, LD < 50%) were considered as instrumental variables (IV) to ensure stable associations between IV and exposure (here, gene expressions). The corresponding SNPs in the CABG genomics cohort were not associated with outcome (here, troponin) to avoid potential pleiotropy. Multivariate MR (M-MR) was applied to avoid potential pleiotropy by the other genes. **B**. Genetic prediction of eGenes at post-ischemia condition with a significant causal effect on troponin levels, (p-value < 0.005). The cell types with the maximum number of counts are provided, where CMC stands for cardiac myocyte and EC for endothelial cell. GBA1LP did not pass the quality control in single-cell analysis. **C**. The boxplots of the two post-ischemia-specific eGenes with causal effect on troponin levels. The normalized expressions at post-ischemia minus baseline for each individual were compared across genotypes of the top cis-eQTL, (x-axes).

We used the summary statistics of the post-ischemia eQTL from the TRANSCRIBE cohort and data from the CABG genomics cohort, (Fig. 3A and Methods). To satisfy the lack of pleiotropy assumption in the MR technique, the genetic variants considered as instrumental variables (IV) were not associated with troponin levels (i.e., outcome), *p*-value > 5E-3, (Supplementary, GWAS Manhattan and locus zoom plots).

We identified 10 genes for which expression levels were causally associated with troponin levels (*p*-value < 0.005) (Fig. 3B), the details of the analysis for the other genes are provided in (Table S6). The maximum counts for these 10 genes have occurred mostly in the CMC cell type. We found that the two genes *FRAT1* and *USP49* were post-ischemia-specific eGenes in the TRANSCRIBE cohort (Fig. 3C), and the remainder were common eQTL in both baseline and post-ischemia conditions.

In the next section, we show that *CFAP161* shares loci signal with MI GWAS; in addition, *TYW1, TMEM80, USP49, FLG,* and *GBAP1* were differentially expressed in human (*p*-value < 8E-3) in which *TYW1* and *TMEM80* were also differentially expressed in mouse (*p*-value < 0.01).

### Genes sharing regulatory (eQTL) and disease-associated (GWAS) signals.

**Figure 4:**
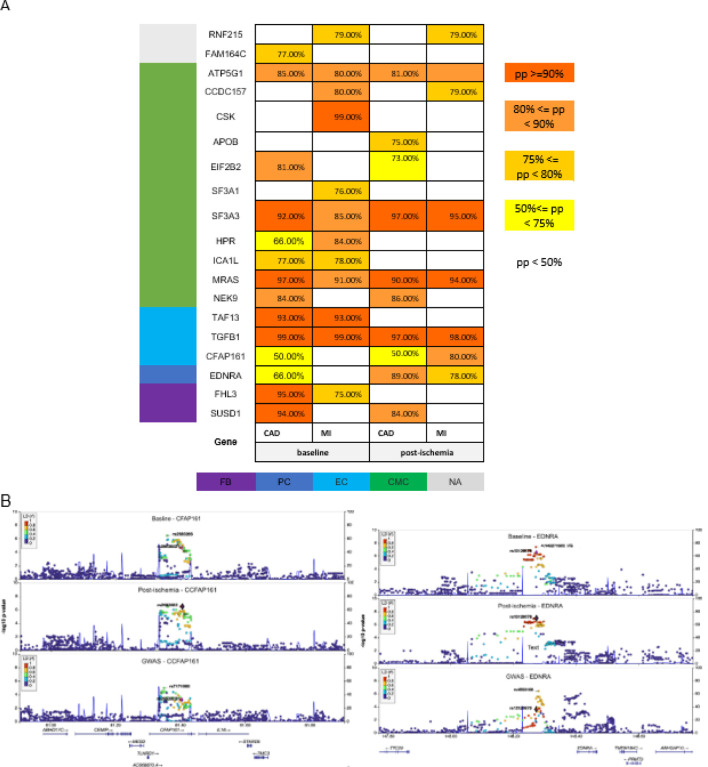
Colocalization of cis-eQTL with CAD/MI GWAS associations. **A**. A heatmap shows all cases with strong evidence of colocalization (PP is written in each block) between cis-eQTL of corresponding genes (eGenes) in rows and GWAS hits associated with CAD and MI in columns at the two conditions. The colors in the column on the left side of the heatmap indicate cell types in which the maximum number of counts has occurred. The maximum counts have occurred mostly in the CMC cell type. CMC stands for cardiac myocyte, FB for fibroblast, EC for endothelial cell, and PC for pericytes. The genes *RNF215* and *FAM164C* did not pass the quality control in single-cell analysis. **B**. Reginal plots of two colocalized genes with GWAS show eQTL association with gene expression of *CFAP161* and *EDNRA* at baseline and post-ischemia conditions and GWAS associations with MI. The minus log10 *p*-value is plotted on Y-axes for all SNPs located within the 1-Mb flanking the gene. The colors of the dots indicate the LD correlation with the top consistent with the cell type estimation in all bulk RNA samples using xCell^18^, (Supplementary).

Using a complementary approach to assess whether genetic regulation of gene expressions can contribute to the risk of CAD/MI, we performed colocalization analysis between eQTL of the two conditions and CAD/MI GWAS summary statistics^11^, (Method). Using the package *coloc*^15^, we estimated posterior probability (PP) for the hypothesis that there is a single causal variant shared between the two different traits (gene expressions and CAD/MI). PP >= 75% is considered strong evidence of a variant influencing both gene expression levels and GWAS traits. We identified 19 genes in both baseline and post-ischemia that may share the same causal variant with CAD/MI, (Fig. 4A).

Among colocalized genes at post-ischemia condition, the genetic predictions of three genes *CFAP161, MRAS,* and *ICA1L* showed a causal effect on troponin levels (p-value < 0.002, 0.01, and 0.02 respectively) with *ICA1L* having a negative impact on troponin levels. Among colocalized genes, *APOB, TGFB1, SF3A3, NEK9, ICA1L, EIF2B2, MRAS, CCDC157, RNF215,* and *FHL3* were differentially expressed in human data, which *TGFB1* and EIF2B2 were also differentially expressed in mouse data. The regional plots of *CFAP161* and *EDNRA* are provided, (Fig. 4B). *EDNRA* is colocalized in post-ischemia condition at (PP > 75%) and in baseline at (PP > 50%). Colocalized genes at the level PP > 50% are provided in (Supplementary, Table S7).

### Single-cell RNA-seq.

**Figure 5:**
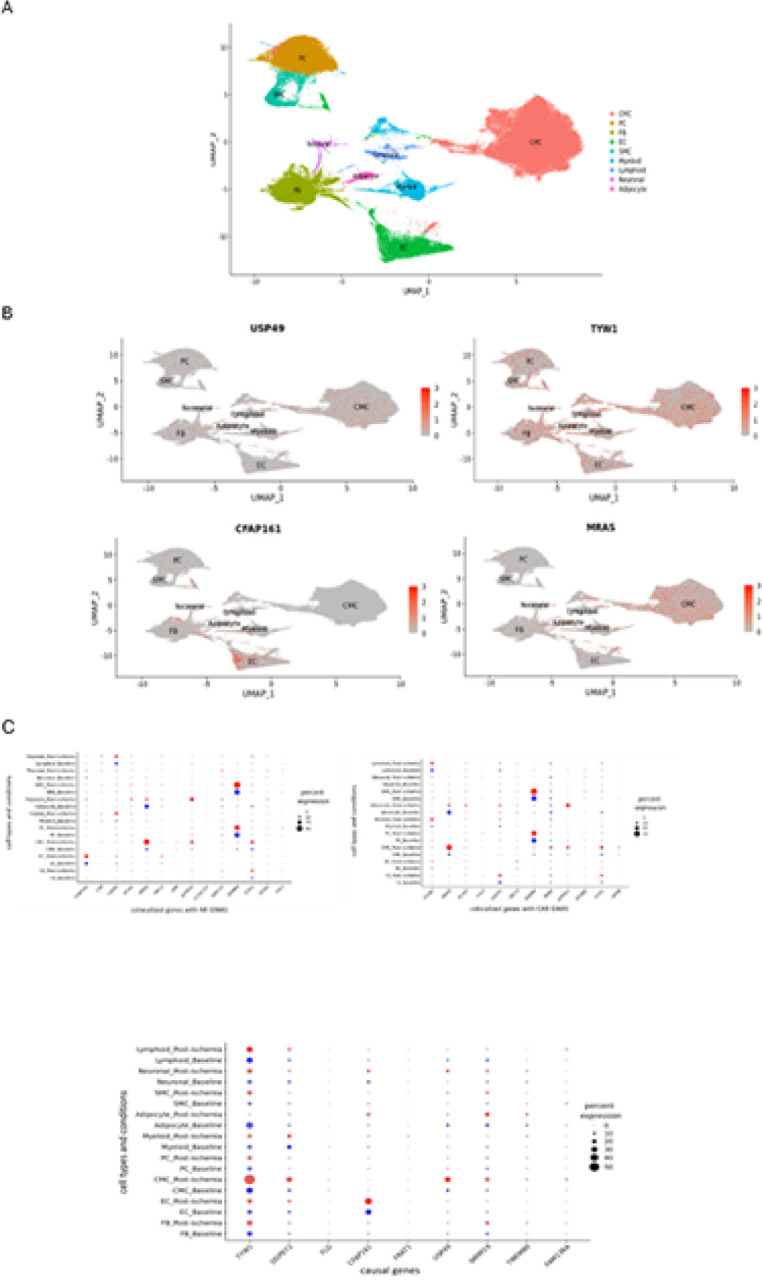
Single-cell RNA sequencing. **A**. The UMAP cluster of all genes. **B**. The UMAP cluster of some causal and colocalized genes. As examples, we see that *TYW1* was expressed in almost all cell types and with a comparatively higher expression in CMC cell type whereas *CFAP161* was predominantly expressed in EC with lower expression observed in other cell types. **C**. The dot plots comparing baseline and post-ischemia expressions in different cell types: the top dot plots represent the genes that share regulatory loci with MI and CAD GWAS (colocalized genes) from Fig.4A and the bottom dot plot represents the genes causally associated with troponin levels from Fig. 3B. As examples, the causal and colocalized gene *CFAP161* has a higher expression in EC compared to other cell types which is slightly higher at post-ischemia condition compared to baseline. The expression of the causal gene *TYW1* is higher at the post-ischemia condition compared to baseline in the CMC cell type and almost equal at both conditions in the other cell types. Similarly, the colocalized gene *MRAS*has a higher expression at post-ischemia condition in the CMC cell type but a lower expression in the adipocyte cell type. The colocalized gene *EDNRA* has a higher expression at the post-ischemia condition in smooth muscle cell (SMC).

We performed the RNA single-cell sequencing on 4 randomly selected samples of the TRANSCRIBE cohort in both baseline and post-ischemic conditions. We clustered the expressions using Seurat 4.1.1^16^ and visualized them using UMAP Cell types were annotated for each cluster based on marker genes from the human cell atlas (HCA)^17^. The gene expressions occurred mostly in cell types of CMC, EC, FB, and PC (Fig. 5), which is consistent with the cell type estimation in all bulk RNA samples using xCell^18^, (Supplementary).

### Genes expressed differently after exposure to ischemia in humans and a comparison with mice.

Using a linear mixed model, we performed differential gene expression analysis and compared the transcriptional expression at baseline and post-ischemia conditions in the TRANSCRIBE cohort, (Supplementary, Table S8). In total, 40 genes were differentially expressed (FDR < 0.05) with absolute log2 fold change (|logFC|) >= 1.5, in which 11 were upregulated (Table 1).

We also leveraged an ischemic myocardium-mouse model to explore the similarity between humans and mice in transcriptional response to ischemia. There were 3 groups of mice based on exposure duration to ischemia: group1 was not exposed to ischemia, group 2 was exposed to ischemia for 30 minutes, and 1 hour and a half reperfusion, and group 3 was exposed to a permanent LAD ligation for two hours (Methods).

**Figure 6:**
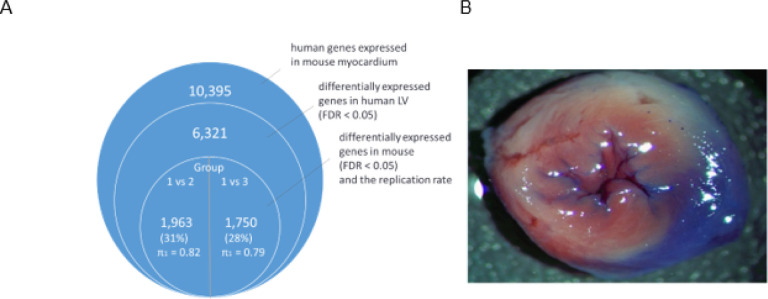
The comparison of human and mouse LV gene expressions. To explore the similarity of humans and mice in terms of LV transcriptional response to ischemia, three ischemic myocardium-mouse models were considered based on exposure to ischemia: group 1 was not exposed to ischemia, group 2 was exposed to ischemia for 1 hour and half reperfusion, and group 3 was exposed to a permanent LAD ligation for two hours. **A**. Differentially expressed Human genes in mouse models. **B**. A picture of ischemic mouse myocardial tissue.

In total, 10,395 human genes were expressed in mouse myocardium among which 6,321 were differentially expressed in human LV (FDR < 0.05), and of these genes, 1,963 (31%) were differentially expressed in mice group 1 vs 2, and 1,750 (28%) in group 1 vs 3, (Supplementary, Tables S9–10), where the replication rate of differentially expressed human genes in mice using π_1_ was 0.82 and 0.79, respectively, (Fig. 6).

## Discussions

The two genes *USP49* (Ubiquitin Specific Peptidase 49) and *FRAT1 (FRAT* regulator of Wnt signaling pathway 1) were post-ischemia-specific eGenes in the TRANSCRIBE cohort (eQTL *p*-value < = 7E-7), which were identified with inverse and direct causal relationships with myocardial injury, respectively. *USP49 was* differentially expressed (*p*-value = 5E-5). It was previously identified that *USP49* inhibits ischemia–reperfusion-induced cell viability suppression and apoptosis in human AC16 cardiomyocytes through DUSP1-JNK1/2 signaling^19^. So, the regulatory function of the gene *USP49* is to promote the protective effects of *DUSP1* on the heart during ischemia-reperfusion injury. This aligns with our findings that *USP49* reduces the level of troponin in the blood (genetically predicted expression of *USP49* had a negative impact on troponin levels). *FRAT1* is involved in Wnt signaling pathway which is regulated after myocardial ischemia to facilitate angiogenesis by ZNF667^20^. *ZNF667* shows inhibitory effects on canonical Wnt pathway-related genes including *FRAT1*^21^.

*CFAP161* (Cilia and Flagella Associated Protein 161) was identified here as an eGene with a causal effect on troponin levels and shares loci with MI GWAS. This gene is associated with the structure and function of cilia and flagella, which are structures found on the surface of many types of cells. These structures have various roles in cellular processes, including the movement of fluids and particles^22^. We observed the higher expression of this gene in endothelial cells which have one cilium that may be important in vascular function/disease; however, limited information is available about the specific functions or associations of this gene from the vascular perspective. A functional study would have to be done in which the gene transcript is identified in the endothelial cell, and the consequences of its knockout assessed.

Another interesting gene that *is* worth pursuing functionally is *TYW1* which is related to S-adenosylmethionine metabolism and guanosine methylation reactions^23^. *TYW1* was identified here as an eGene with a causal effect on troponin and differentially expressed in humans and mice. This gene encodes an enzyme known as “tRNA-yW synthesizing protein 1” which plays a role in the modification of transfer RNA (tRNA) molecules^24^. The yW modification stabilizes codon-anticodon interactions during translation, which helps prevent errors and ensures the fidelity of protein synthesis. Defects or mutations in the *TYW1* gene can potentially lead to issues with tRNA modification and affect protein synthesis.

We identified the gene *GAPDH* (Glyceraldehyde 3-phosphate dehydrogenase) as a post-ischemia specific eGene (*p*-value = 2E-6), differentially expressed in humans (*p*-value = 0.001), and the *p*-value of differential expression in mice was 0.08. *GAPDH* is an enzyme involved in breaking down glucose to obtain energy. The nuclear *GAPDH* forms a protein complex with p53 and enhances p53 expression and phosphorylation which is important in regulating glutamate-mediated neuronal death, and the interfering peptide in vivo protects against ischemia-induced cell death in rats subjected to tMCAo^25^.

The findings in this study, such as the genes discussed above offer the potential for the development of novel biomarkers for early detection and risk assessment in individuals with or at risk of heart ischemia. For example, monitoring the expression of *USP49,* which has previously been associated with protection against ischemia-reperfusion-induced cell damage, could serve as an indicator of the heart’s resilience to ischemic stress. Furthermore, understanding the functional roles of these genes, such as *CFAP161* in endothelial cells or *TYW1* in S-adenosylmethionine metabolism, provides opportunities for targeted interventions.

One limitation of this study was that the model considered for generating data may not reflect clinical ischemia due to the unique setting of bypass conditions, such as cold cardioplegic arrest and a lack of obstructive coronary disease. Therefore, the findings may not be generalized to typical atherosclerosis-related ischemia. However, the model shares a commonality with ambulatory MI by increased cardiac biomarker release. The model also tells us about eQTL change and the type of ischemia-induced during bypass surgery, which itself is a major clinical problem. The other limitation of this study is the sample size of 118 individuals for eQTL analysis, therefore, we limited the study to cis-eQTL. However, the strength of this model lies in having tissue samples from the same individuals under the two conditions baseline and post-ischemia. Another strength of this study is using summary data of the eQTL analysis to impute gene expressions into an independent cohort with relatively a larger sample size, of 2,371 patients. The findings here open the door to future interventional genetic studies in mouse models and exciting possibilities for translating genetic insights into clinical practice.

## Methods

### Patient population and clinical variables. The study protocols were approved by the Institutional Review Board and all patients provided written informed consent 26.

#### Human LV Tissue Samples.

Patients (of Caucasian ancestry) presenting for elective aortic valve replacement surgery by a single surgeon at Brigham and Women’s Hospital were enrolled in this study (https://clinicaltrials.gov/ct2/show/NCT00985049). The patients provided written informed consent. Patients were deemed to have CAD if obstructive lesions were noted on left heart catheterization before aortic valve surgery requiring concomitant revascularization of at least one vessel. Punch biopsies (~3–5μg total RNA content) were obtained intra-operatively from the site of a routinely placed surgical vent in the anterolateral apical left ventricle at the initiation of CPB (baseline sample) and again after an average of 81.7 ± 27.9 minutes of aortic cross-clamp time (post-ischemia). During aortic cross-clamping, the heart was infused with intermittent cold blood cardioplegia (8:1 blood to crystalloid ratio) for myocardial protection.

#### DNA quality control.

For the quality control and preparation of genotype data, DNA extracted from whole blood served as the source material for SNP genotyping, carried out using the Illumina Omni2.5 genotyping array^26^, which includes exome content. Before imputation and subsequent analysis, a rigorous quality control process was applied. Variants or samples exhibiting a missingness rate exceeding 2% or deviating from Hardy-Weinberg equilibrium (HWE) with a p-value less than 0.001 were systematically eliminated using the Plink software (available at https://zzz.bwh.harvard.edu/plink/).

The assessment of sample consistency in terms of genetic data and self-reported sex was conducted using Plink. Following pre-imputation processing employing the Haplotype Reference Consortium (HRC) Checking tool, the genotypes were uploaded to the Michigan Imputation Server, accessible at https://imputationserver.sph.umich.edu/index.html#! The imputation process was configured with Eagle284 and the HRC/hg19 reference panel.

Variants characterized by low-quality scores (less than 0.8), a minor allele frequency (MAF) below 0.05, or those not adhering to HWE were systematically removed from the dataset. In cases where pairs of samples exhibited a probability of relatedness exceeding 25%, one of the samples was selected for further analysis.

#### Bulk RNA Sequencing.

Tissue samples were immediately placed in RNAlater^®^ (Ambion, Life Technologies, USA), and were stored after 48 hours at +4°C at −80°C until the extraction of RNA. Total RNA was isolated with Trizol. The RNA quality was assessed using the Agilent Bioanalyzer 2100 (Agilent) with no samples being excluded for poor quality. Library preparation and sequencing have been described previously^27^. By performing 1–2 washings of RNA annealed to poly-T oligos beads (Invitrogen), ribosomal RNA was removed and then, using random hexamers (Invitrogen), reverse transcribed. Using Pol I and RNA-ase H, Double-stranded DNA (dsDNA) synthesis was performed. Short fragments were purified with QiaQuick PCR extraction kit (Qiagen) and resolved with EB buffer for end reparation and poly(A) addition after that, ligated with sequencing adaptors for cluster generation and sequencing on the Illumina HiSeq 2000 (Illumina, San Diego, CA). Since samples were analyzed at different times, different read lengths were employed: initially, single-end reads and then, paired-end reads ranging from 36 to 100 base pairs.

#### Alignment of RNA sequencing.

Using Spliced Transcripts Alignment to Reference, sequences were aligned to the human genome (GRCh37) after adapter and quality trimming. Next, genes with low counts (count per million > 1 for fewer than 12 patients, equivalent to 10% of samples, were removed (N_baseline_ = 8,077 genes, N_post-ischemia_ = 7,805 genes). Focusing on the union of the genes in chromosomes 1–22 that passed filters (separately applied for each condition), 14,990 genes were left to normalize. We applied TMM across samples and then applied an inverse normal transformation to each gene. The pre-processing was carried out independently for each condition.

#### Cell type deconvolution analysis.

We estimated the cell types in the ischemic heart left ventricle using XCell^18^. To better estimate cell type enrichments, we combined our ischemia heart left ventricle samples with >17k samples from the GTEx project across 56 tissues of the human body. We benchmarked the results of our enrichment estimations with the estimations of 7 cell types (adipocytes, epithelial cells, hepatocytes, keratinocytes, myocytes, neurons, and neutrophils) available in the GTEx portal (Supplementary).

#### cis-eQTL mapping.

A total of 6,994,645 SNPs were available from 118 individuals for the cis-eQTL analysis. For each condition, we performed a cis-eQTL analysis considering SNPs +/− 1 Mb from each tested gene. We applied a linear regression model using QTLtools^28^, where we used the adaptive permutation model with the default parameter settings. We included sex, age, first three genotype principal components, and aortic cross-clamp duration (only for post-ischemia eQTL analysis) as covariates in the model. We considered only the first three genotype principal components because the data were genetically homogenous, and the variation explained by the first principal component was less than 1%. In addition, we applied the Probabilistic Estimation of Expression Residuals (PEER) method to the gene expression data in each of the conditions to generate factors to account for technical and other confounding sources of expression variation, from known or unknown sources^29^. Based on maximizing cis-eGene discovery^13^, we included 20 and 15 PEER factors for the baseline and post-ischemia eQTL analyses, respectively. The covariates with a high correlation to PEER factors were not included in the model. The gene expression variance captured by PEER factors from each condition was correlated with known technical and biological covariates recorded for each sample. The covariates that were most consistently associated with PEER factors include factors related to sequencing quality control metrics and cell type proportions. The quantifications were performed using the variancePartition package in R^30^, (Supplementary).

The beta distribution-extrapolated empiricalp-values were used to identify genes with at least one significant cis-eQTL. Then, we applied FDR threshold < 0.05 using the Benjamini-Hochberg method to select eGenes

#### cis-eQTL replication.

To quantify the extent of eQTL sharing across conditions or replication in independent datasets, we used Storey’s π_1_ statistics^12^. The π_1_ statistics considers the full distribution of association *p*-values (from 0 to 1) and computes their estimated π_0_, the proportion of eQTL that are truly null based on their distribution. Replication is reported as the quantity π_1_ = 1 - π_0_ that estimates the lower bound of the proportion of truly alternative eQTL.

To replicate cis-eQTL across conditions (within the study), we selected the most significant variant per eGene and queried its corresponding *p*-value in the other condition, using the resulting nominal p-values to estimate π_1_. In addition, we replicated our eQTL with GTEx eQTL, V8 release (https://gtexportal.org/home/datasets). We estimated π_1_ eQTL in GTEx V8 heart left ventricle (HLV) and heart atrial appendage (HAA), as well as the lung. We matched genes using HGNC Multi-symbol checker (https://www.genenames.org/tools/multi-symbol-checker/) and used liftOver from the R package rtracklayer to map our dataset hg19 genomic coordinates to GTEx V8 hg38 coordinates. We then identified matching SNP-gene pairs in the “All variant-gene cis-eQTL associations” HLV, HAA, and Lung from the GTEx download site (https://www.gtexportal.org/home/datasets).

#### Two-sample MR to identify the causal genes of troponin as a biomarker of cardiac injury.

To identify the causal genes of troponin, we used data from the CABG genomics cohort^31^ and eQTL summary statistics at the post-ischemia condition from the TRANSCRIBE cohort, proceeding through the following steps:

1. **Prediction of gene expression levels in the CABG genomics cohort:** We focused on 1,728 eGenes at the post-ischemia condition. From CABG Genomics, we first selected all SNPs corresponding to cis-eQTL of each gene (FDR < 0.05) in the TRANSCRIBE cohort, resulting in 657,807 genetic variants nominally associated with genes. We clustered the genetic variants of each gene based on LD (r^2^ ≥ 0.5) using a hierarchical clustering approach^32^. Within each cluster, we selected the eQTL with the largest effect size (beta divided by standard deviation) to represent each cluster. For each cluster, we allocated a score based on the number of SNPs in the cluster. Using Equation (1)^14^, we predicted the level of expression of each gene for the individuals in the CABG genomics cohort (Fig. 3A),
(1) $$\hat{G}=\sum_{i=1}^{k}w_{i}*\beta_{eSNP_{i}}*SNP_{i,\text{GWAS}},$$
  where $\hat{G}$ stands for the predicted expression levels of a gene for the individuals; *k* stands for the number of clusters, and *w*_*i*_ is the score of cluster *i, SNP*_*i,GWAS*_ is the representative of cluster *i* which the corresponding eQTL has the largest effect size on the gene, and $\beta_{eSNP_{i}}$ stands for the eQTL effect on the corresponding gene.
  Selecting a proxy from each cluster and considering the weight prevents spurious estimates and inflated Type 1 errors when using too many genetic variants in the analysis or highly sensitive estimates due to ignoring most data^14^. However, it may reduce the impact of variants on shorter haplotypes.
2. **Investigating causal relationships between predicted genes and troponin:** We estimated the effect of genes (i.e., exposure/explanatory variables) on troponin (i.e., outcome) using multivariable models. If a variant exhibits pleiotropy through some other genes, including those genes as additional explanatory variables mitigates bias by jointly estimating the causal effects of all genes on troponin^33^.
  Of the 2,371 individuals in the CABG genomics cohort, 1,744 had records on clinical covariates and troponin levels. Therefore, this step was performed on 1,744 individuals. Since our focus is on the 1,728 eGenes at post-ischemia condition, we performed 10 multivariable models following the recommendation that the sample size must be 10 times the number of independent variables^34^. We performed models while controlling for the CABG cohort covariates (age, gender, hospital, preoperative HMG Co-A reductase inhibitor use, Coronary stenosis, CPB time, pre-operation creatinine), and assuming an additive effect of gene expression on troponin, Equation (2), (Fig. 3A),
(2) $$T=\alpha +\beta_{1}{\hat{G}}_{1}+\beta_{2}{\hat{G}}_{2}+\cdots +\beta_{n}{\hat{G}}_{n}+\Lambda E+e$$
  where *T* stands for log-transformed troponin levels of 1,744 individuals, $\hat{G}$ is the predicted gene expression, *n* is the number of genes in the multivariable analysis, *E* is a vector of the covariates, and finally, *β* and *Λ* are the effect/coefficient of genes and covariates respectively. The resulting *p*-values were adjusted for multiple testing using Bonferroni correction, p-value ≤ 0.005 = 0.05/10 to account for the 10 multivariable models. The genes with significant effects on troponin at level (*p*-value <0.1) using multivariable MR are provided in (Supplementary, Table S11).
3. **Assessing the MR assumptions:** To identify the causal genes of troponin levels, we satisfied the MR assumptions while predicting the gene expressions. The genetic variants considered as IVs were cis-eQTL (FDR < 0.05) and assumed to have a stable relationship with genes. In addition, we predicted each gene using multiple genetic variants with LD < 50% to increase the stability of the IV-gene relationship (the reason for LD < 50% is to avoid overfitting). Furthermore, we applied multivariable MR (fitting troponin as an outcome on multiple gene expressions) to reduce the bias due to pleiotropy via other genes^33^. Moreover, we used the eQTL summary data from TRANSCRIBE cohort and predicted genes in the CABG genomics cohort with biologically independent individuals. There is a low chance for the two independent cohorts to have the same confounding environmental factors. Finally, the corresponding SNPs of the IVs in the CABG genomics cohort, which correspond to the eQTL, were not directly associated with the outcome (p-value > 0.005) in troponin GWAS with 2,371 individuals. This means in the MR analysis; we focused on indirect associations of SNPs and troponin through gene expressions to identify if gene expressions and troponin are causally related.

#### Colocalization of cis-eQTL with coronary artery disease.

To assess if the cis-eQTL identified in this study (both baseline and post-ischemia conditions) and CAD/MI share the same causal variants, we performed colocalization analysis between the LV cis-eQTL summary statistics and the CAD/MI GWAS summary statistics from the CARDIoGRAMplusC4D Consortium^11^.

The genomic co-occurrence of eQTL and GWAS signals can be due to chance or due to the two traits sharing the same underlying causal variants. To distinguish between these alternatives, we applied a Bayesian method implemented in *coloc*^15^ and estimated the probability that the association signals are due to the same causal variants. The *coloc* estimates the posterior probability of both traits being associated and sharing a causal variant single. A posterior probability > 75% is considered strong evidence of the variant influencing both gene expression levels and GWAS traits.

#### Differential expression analysis:

We performed a differential expression analysis to evaluate changes in the gene expression levels after exposure to ischemia. Baseline and post-ischemia samples were modeled as within-individual comparisons by adding the individual identifier as a random effect and pairing term. In addition, the time between the baseline sample and the post-ischemia sample was adjusted after mean centering. We applied the following linear mixed model:

(3) $$G=\alpha +\beta_{1}\text{ condition }+\beta_{2}\text{ mean\_ischemic\_}{\text{time}}_{0}+\text{ Study. ID }+e$$

where *G* is the gene expression level, *β*_*i*_*s* indicate a fixed-effect regression coefficient, Study. ID is the random effect associated with each individual, and *e* is the error term.

The gene expression at baseline and post-ischemia for 118 samples was first normalized and adjusted for the counts non-normality with the *voomWithDreamWeights* function and then a linear mixed-model was fitted using the *dream* function^30^. The *eBayes* function from the *limma* R package (v 3.50.3)^35^ was then applied to the “condition” coefficient to estimate a moderated t-statistic for each gene fold-change between baseline and post-ischemia. FDR < 0.05 using the Benjamini–Hochberg method was applied to account for the large number of tests.

#### Single-cell RNA-sequencing.

Out of 118 LV tissue samples, four were considered for single-cell RNA-sequencing at the two conditions, baseline and post-ischemia conditions, to identify the cell types with the maximum number of reads mapped on a given gene and also compare baseline and post-ischemia expressions in different cell types for the findings in this study.

#### Clustering single-cell RNAseq data.

Data integration was performed on the four LV samples across the baseline and post-ischemic conditions using Harmony algorithm^36^ in Seurat 4.1.1^16^. The integrated and normalized dataset was then subjected to clustering at 0.6 resolution, which sets the granularity of clusters. Seurat uses the Louvain algorithm, to iteratively group cells together to cluster the cells. Non-linear dimensional reduction technique, UMAP was used to visualize and explore these datasets. Cell types were annotated for each cluster based on marker genes. The identified marker genes were used to annotate clusters as specific cell types. Human Cell Atlas (HCA)^17^ was the reference database used here.

#### Single-cell differential expression analysis.

To evaluate changes in the expression levels of genes in each cluster of the cell type of interest at baseline and post-ischemia conditions, we performed cell type-wise differential expression analysis using DESeq2 package in R (version 4.2.1)^37^, which is based on negative binomial generalized linear models (GLM). We compared the gene expression of baseline and post-ischemia counts of the 4 samples. Metrics for aggregation across cells in a sample were acquired from the integrated dataset. The data was split and aggregated by cell type, i.e., for each cell type e.g., myocyte, fibroblast, etc.

#### Murine Ischemia Reperfusion and MI Models.

All procedures were approved by the University of Massachusetts Medical School IACUC (Docket #A-1600-13-17). 10-week-old wild-type male mice were purchased from Jackson Labs (C57Bl6/J). We performed an ischemia-reperfusion experiment in 3 groups using previously described methods^38,39^. Group 1, included 6 mice as control/sham, not exposed to ischemia, group 2, included 6 mice that were exposed to ischemia for 30 minutes, and 1 hour and half hours of reperfusion and group 3 included 8 mice exposed to a permanent LAD ligation for two hours. Depilatory cream was applied to the chest of each mouse the evening before surgery. Mice were induced with 3% isoflurane and oxygen with a flow rate of 0.4 L/min. Mice were then placed on a heated surgical platform, maintained with 2% isoflurane in 100% oxygen with a nosecone, with limbs gently affixed to the surface with surgical tape. A surgical platform was used to keep mice warm and central temperature was monitored with a rectal probe, maintaining a body temperature of 37°C. The chest was shaved and prepped with betadine and alcohol. Mice were tracheally intubated and ventilated with 2% isoflurane in oxygen with a flow rate of 0.4 L/min. A chest incision was made at the fourth intercostal space, thoracic muscles were dissected down to the ribcage. The thoracic cavity was opened with surgical scissors into the intercoastal space. Retractors were used to pull open the incision. The pericardium was incised and pulled back along the left lung. A dissection microscope was used to locate the LAD. The heart was gently held in place with a surgical swab. A 6–0 silk suture was passed under the LAD approximately 2 mm distal to the apex of the left atrium. For animals in the IR group, a double knot was made, over a 3 mm piece of PE-10 tubing to compress the LAD for 30 minutes of ischemia. In group 3, the LAD was ligated directly with a knot. Confirmation of LAD occlusion was made via confirmation of blanching of the anterior wall of the LV seconds after ligation. After 30 minutes of ischemia, the PE-10 tubing was removed and animals were re-perfused for 1.5 hours. The LAD was then re-occluded and 10% Phthalo Blue (Sigma) was perfused via the aorta to stain the heart. The heart is then excised and washed in 30 mM KCL and frozen at −20 °C. In the infarct group, the ligature was left in place for two hours, Phthalo blue was administered and then the animals were euthanized. In the sham/control group, animals were euthanized after two hours with their chests open.

When ready, hearts were sectioned into 1 mm slices and incubated with 2% TTC (Sigma) at 37°C for 40 minutes. The infarct area is white, the ischemic area is blue, and the viable tissue is red. Stained slices were fixed with 10% formaldehyde overnight.

Total RNA was isolated from snap-frozen sections using Qiagen mini RNA kits (Qiagen). RNA quality was assessed using an Agilent 2100 Bioanalyzer. Then, RNA sequencing was performed. The libraries were built with a stranded strategy protocol and Ribo-Gone was used to remove rRNA before cDNA synthesis. Libraries were created and samples were sequenced by the University of Massachusetts Medical School Deep Sequencing Core. This work was completed in part with resources provided by the University of Massachusetts’ Green High-Performance Computing Cluster (GHPCC). The RNAseq was aligned using Spliced Transcripts Alignment to Reference and the *limma* R package was used for differential gene expression analysis between groups 1 verses 2 and 3 similar to human data.

## Figures and Tables

**Table 1.** Upregulated genes after exposure to ischemia

Gene	logFC	p-value	adj p-value
FOSB	2.9	9.16E-17	5.20E-15
FOS	2.5	5.59E-17	3.30E-15
CXCL8	2.0	3.75E-14	1.12E-12
HBA2	1.8	1.31E-27	1.51E-24
S100A8	1.8	2.92E-19	2.99E-17
HBA1	1.8	2.90E-28	4.36E-25
HBB	1.7	2.04E-28	3.42E-25
TREM1	1.6	4.54E-16	2.14E-14
S100A9	1.5	4.18E-18	3.23E-16
SOCS3	1.5	4.43E-12	8.05E-11
CXCL2	1.5	1.56E-17	1.08E-15

(|log(FC)| >= 1.5 and FDR < 0.05).

## Data Availability

All data generated during this study will be available through Harvard Dataverse, https://doi.org/10.7910/DVN/O6DKJM

## Abbreviations

CPB: cardiopulmonary bypass
CAD: coronary artery disease
eQTL: expression quantitative trait loci
eGene: the gene with at least one significant cis-eQTL
FDR: False Discovery Rate
GWAS: genome-wide association study
LV: left ventricle
MI: myocardial infarction
MR: Mendelian randomization
scRNA-seq: single-cell RNA sequencing
SNP: single nucleotide polymorphism
